# Epigenetic Editing: On the Verge of Reprogramming Gene Expression at Will

**DOI:** 10.1007/s40142-016-0104-3

**Published:** 2016-10-01

**Authors:** David Cano-Rodriguez, Marianne G. Rots

**Affiliations:** Epigenetic Editing Research Group, Department of Pathology and Medical Biology, University Medical Centre Groningen, University of Groningen, Hanzeplein 1, 9713 GZ Groningen, The Netherlands

**Keywords:** Epigenetics, Gene expression, Chromatin, Zinc finger proteins, TALE, CRISPR-dCas

## Abstract

Genome targeting has quickly developed as one of the most promising fields in science. By using programmable DNA-binding platforms and nucleases, scientists are now able to accurately edit the genome. These DNA-binding tools have recently also been applied to engineer the epigenome for gene expression modulation. Such epigenetic editing constructs have firmly demonstrated the causal role of epigenetics in instructing gene expression. Another focus of epigenome engineering is to understand the order of events of chromatin remodeling in gene expression regulation. Groundbreaking approaches in this field are beginning to yield novel insights into the function of individual chromatin marks in the context of maintaining cellular phenotype and regulating transient gene expression changes. This review focuses on recent advances in the field of epigenetic editing and highlights its promise for sustained gene expression reprogramming.

## Introduction

Epigenetics is the study of heritable yet reversible changes in gene expression, which are independent of the underlying DNA sequence. Although all cells within an organism contain the same DNA, there are many different cell types, making the various tissues and organs, present. Many genes are constantly activated or repressed leading to these different phenotypes [1]. This epigenetic gene regulation is mediated by several mechanisms that work together in order to determine the cell type-specific patterns of expression. The organization of DNA and histones into chromatin is an important aspect in gene regulation, through which the access of transcription complexes to the DNA can be regulated [2]. Chromatin is organized in nucleosomes (protein octamers, generally consisting of two copies of each core histone H2A, H2B, H3, and H4, where 147 base pairs of DNA is wrapped around) and a linker histone (H1). Higher-order folding of the nucleosomes can result in many chromatin states, with the simplest classification being less condensed, active euchromatin or highly condensed, silent heterochromatin [3].

Next to maintaining mitotically stable expression patterns, chromatin controls DNA accessibility through, for instance, post-translational modifications (PTM) of the histone tails or modification on the DNA such as methylation [4]. These modifications can directly or indirectly influence chromatin structure by modulating DNA–histone interactions and form docking sites to facilitate recruitment of proteins to the chromatin [5]. This form of epigenetic regulation is important for the maintenance of cell identity and therefore it is implicated in processes such as proliferation, development, and differentiation [6]. The patterns of histone PTMs that occur on the histone tails form a so-called histone code that can be deciphered by other proteins. These proteins can alter the structure of higher-order chromatin and in turn recruit other effector molecules [7, 8].

For several years, it has been under heavy debate whether chromatin marks are the cause or mere consequence of gene expression or repression [9–11]. Most studies addressing chromatin and RNA expression are based on statistical associations of various chromatin marks with expression levels of the genes [12–14]. Such studies firmly established associations between, for example, H3K4me and active gene expression, or H3K9me and H3K27me and gene repression. However, it is worth mentioning that correlation does not necessarily imply causation. Epigenetic research has long been hindered by the lack of experimental methods that would allow the targeted manipulation of chromatin marks in living cells. Most of the studies have used mutational approaches and pharmacological inhibition to alter epigenetic marks, but this has global and non-chromatin effects [15, 16]. Nevertheless, using these techniques scientists have been able to provide further support that loss of chromatin modifiers causes strong phenotypes, which are often interpreted as a consequence of transcriptional deregulation, although the cellular effects might very well be established through changes in non-chromatin targets [17].

An elegant approach to actually rewrite epigenetic modifications at a known locus was the targeting of epigenetic effector domains to reporter genes. Early research made use of synthetic protein–DNA-binding approaches (e.g., Gal4, LacR), or fused existing human DNA-binding domains to (parts of) epigenetic enzymes (e.g., MLL, NF-kB). Currently, it is feasible to target epigenetic effector domains to any given genomic locus (referred to as “epigenetic editing” [18•], making it experimentally possible to modify individual chromatin marks at a defined locus and chromatin context [19, 20].

The goal of such epigenetic editing is to rewrite an epigenetic mark at any locus at will, and eventually modulate the expression of endogenous genes. In order to rewrite a gene’s epigenetic signature a (catalytic domain of a) writer or an eraser can be targeted to the given locus by fusing it to a programmable gene-specific DNA-binding domain (DBD) [21–29]. Induced epigenetic changes can be determined by, e.g., chromatin immuno-precipitation (ChIP) or bisulfite sequencing and the actual effect of targeting epigenetic enzymes on gene expression can be assessed by measuring gene expression levels of genes that are in close proximity of the DBD recognition site. In this review, we summarize recent epigenetic editing reports using different DNA-binding platforms and several activators, repressors, or epigenetic enzymes targeted to endogenous loci.

## Gene Targeting Platforms

In recent years, the molecular biology field has developed three protein systems to design domains with predetermined DNA sequence-binding specificity. C2H2 zinc finger proteins (ZFPs) were the first example of modular and predictable DNA recognition proteins and a few research groups worldwide, including ours [30–33], exploited this first generation system to demonstrate its power to modulate expression of any given gene of interest. These early studies were exploiting non-catalytic domains to modulate gene expression including, e.g., a viral transcriptional activator (VP16 and its tetramer VP64) [34, 35] or the mammalian repressor KRAB [30, 36]. More recently, a more straightforward programmable recognition domain platform was introduced: the Transcription-Activator-Like Effector (TALE) arrays [19]. Both platforms, however, require the fusion of the effector domain to every newly engineered DNA-binding domain, which is a laborious, expensive, and greatly hampered progress. The introduction of the Clustered Regulatory Interspaced Short Palindromic Repeats (CRISPR) sequences with CRISPR-Associated Protein (Cas) or CRISPR/Cas9 systems has made epigenetic editing available to the wider research community as it consists of two simple modular parts: a sgRNA (which is easy to design and cheap) and its to be recruited counterpart, the protein dCas (allowing a one-time fusion to an epigenetic editor for all possible targets) [37]. Indeed, recent findings clearly indicate the promise of epigenetic editing to reprogram gene expression patterns, and are discussed below.

## ZFPs

ZFPs are among the most common types of DNA-binding motifs found in eukaryotes and are present in many natural transcription factors. They can be engineered to recognize almost any DNA sequence [38]. ZFPs are made of modular zinc finger domains in which each finger consists of ca 30 amino acids containing one α-helix and two β-sheets that are coordinated by a zinc ion, generally with two residues of cysteine and two residues of histidine. Three amino acids on the surface of the α-helix typically contact three base pairs in the major groove of DNA [39]. By linking six ZF domains together, a 6-ZFP can be engineered to recognize 18 base pairs of DNA, which is mathematically unique in the genome [40]. This way, ZFPs can be used to target DNA sequence in the genome. An individual finger domain recognizing a 3 base pair segment of choice is selected from lists of artificially constructed fingers, such as Barbas modules for 5′-GNN-3′, 5′-ANN-3′, 5′-CNN-3′, and a partial 5′-TNN-3′ [41]. For many years, engineering ZFPs was the only approach available to create custom site-specific DNA-binding proteins. Nevertheless, they are expensive, labor intensive to create, and not highly specific. On the other hand, they constitute the smallest of the three currently available platforms. One of the most important rules to designing DNA-binding platforms has been the use of DNAse hypersensitive sites, which mark regions of open chromatin. Interestingly, ZFPs due to their size are able to bind highly chromatinized regions in the genome, in contrast to other platforms [42•]. Additionally, they are presumably less immunogenic due to their similarity to mammalian transcription factors. Currently, engineered ZFPs are available commercially from Sigma–Aldrich (St. Louis, MO, USA), and are the only domains, which have been explored in clinical trials, for over ten years now (Sangamo Biosciences, Richmond, CA, USA).

## TALEs

TALEs are derived from the bacterium species *Xanthomonas.* In host plants, they affect gene expression by binding to promoters of disease resistance-related genes and regulate their expression to facilitate bacterial colonization and survival. TALEs contain 13–28 highly conserved tandem repeats of 33 or 34 amino acid segments; these repeats mostly differ from each other at amino acid positions 12 and 13 [19, 43]. Unique combinations of amino acids at the positions 12 and 13 bind to specific corresponding nucleotides, allowing for gene targeting (for example, NI to A, HD to C, NG to T, and NN to G or A). Like ZFPs, modular TALE repeats are linked together to recognize contiguous DNA sequences. Although the single base recognition of TALE to the DNA allows greater design flexibility than triplet-confined ZFPs, the cloning of repeat TALE arrays presents a technical challenge due to extensive identical repeat sequences. Moreover, their big sizes and immunogenicity likely will hamper their uses in clinical applications. Likewise, DNA methylation has been shown to hamper the binding of TALEs, restricting their accessibility at heterochromatin regions [44].

## CRISPR

The discovery of the CRISPR-Cas system has been one of the most important advances of the century in molecular biology research. CRISPR-Cas originally was identified to act as an immune system in bacteria, but is now largely exploited as a gene-targeting platform because of the ease of the approach. There are at least three different CRISPR classes under development, with type II CRISPR/Cas9 of *Streptococcus pyogenes* being the simplest design, composed of a single endonuclease protein Cas9. CRISPR-Cas9 main function is to detect pathogenic DNA and shred it. Recognition of pathogenic DNA is achieved by incorporating the short host DNA segment in the Cas locus of the bacteria. This DNA is transcribed into a so-called single guide RNAs (sgRNAs) that recognize the host target genomic sequence of approximately 20 bps upstream of a 5′-NGG-3′ protospacer adjacent motif (PAM). The requirement of a PAM sequence slightly limits the targeting freedom of CRISPR/Cas9, occasionally making the use of ZFPs and TALEs more advantageous in cases where no 5′-NGG-3′ sequence is present. Upon binding, the Cas9 nuclease can cleave double-stranded DNA with its RuvC-like nuclease domain and HNH nuclease domain. Keeping the nuclease activity intact thus allows for gene editing by inducing double-stranded DNA breaks and relying on homologous recombination (HR) or non-homologous end joining (NHEJ) for cellular DNA repair. The nuclease domains of Cas9 can be enzymatically inactivated through mutations in the RuvC and HNH domain, thereby creating the nuclease-null deactivated Cas9 (dCas9), e.g., gene expression manipulation purposes. CRISPR offers similar high levels of efficiency to TALEs, and its design and implementation is simpler than that of ZFPs and TALES. However, several concerns have also been raised regarding the specificity of the CRISPR system. Mismatches between the DNA target sequence and RNA molecule are tolerated, increasing the possibility for off-target effects. Additionally, the size and immunogenicity of the Cas9 protein make the clinical application of the system a likely hurdle. These limitations require further exploration. However, this system has opened several opportunities to study a plethora of applications in biology, such as gene expression modulation. Interestingly, the first ex vivo clinical trial using CRISPR for genome editing has been approved recently [45].

## Artificial Transcription Factors

The fusion of transcriptional effector domains to designed DNA-binding domains can induce transcriptional activation or repression when targeted to endogenous genes. The ZFPs were the first to be linked to the transcriptional activator VP16 to create an artificial transcription factor [38, 46]. VP16 is an activation domain from the herpes simplex virus that recruits the RNA polymerase II transcriptional machinery [47]. Later, a tetramer of VP16 domains (VP64) was created and has been linked to several DNA-binding platforms to activate coding and non-coding genes by targeting the promoters and regulatory elements in the genome. However, VP64 does not directly modify chromatin and has been shown to have a transient effect on gene expression [42•]. Nevertheless, it recruits several factors linked to increased chromatin accessibility and the deposition of active histone marks, such as acetylation of the lysine 27 residue of histone subunit 3 (H3K27ac) [48, 49]. Another activator exploited for targeted gene activation is the p65 subunit of the human NF-κB complex, which has been coupled to ZFPs [50], TALEs [51, 52], and dCas9 [53]. Gene induction by these activators can be achieved by targeting both up- and downstream of transcription start sites (TSSs) in promoter regions. However, the activation of gene expression using these proteins has not been very efficient in all cases, depending on the region targeted, and for this reason recruitment of multiple DNA-binding domains to a locus is often required to achieve a robust transcriptional response, especially in the case of dCas9 system.

In order to overcome low efficiency of activation, a new generation of activators have been developed that allow robust gene overexpression in comparison to the original domains. These new activators work by amplifying the recruitment of multiple effectors to a single dCas9-gRNA complex. For example, the SUperNova Tagging (SunTag) system, which recruits multiple VP64 activators to dCas9 in trans, results in stronger activation with a single gRNA [54]. Alternatively, repurposing the gRNA as a scaffold to recruit activators via MS2-targeting has been proven effective: The authors fused several RNA hairpins from the male-specific bacteriophage-2 (MS2) to the 3′end of a sgRNA and fused the MS2 coat protein (MCP), which binds the MS2 hairpin, to VP64, resulting in efficient activation [55]. Similarly, the synergistic activation mediator (SAM) system uses two MS2 hairpins in the sgRNA and fuses MCP to the activators p65 and HSF-1 (Heat Shock Factor 1, responsible for transcribing genes in response to temperature) [56]. This system is used in combination with dCas9–VP64 and showed a significant improvement compared to the other systems. Lastly, the VPR system using three separate activators (VP64, p65, and Rta) has been shown to achieve high levels of expression [53].

Transcriptional repression has also been accomplished by using targeted gene silencing with engineered DNA-binding domains fused to repressors. Targeting of a DNA-binding domain without any effector domain to promoter regions or regions downstream of the transcription start site can silence gene expression by steric hindrance of transcription factors and RNA polymerase [46, 57]. However, gene repression by this method alone generally is not sufficient for robust silencing. Transcriptional repressors, which by themselves possess no catalytic activity but can recruit epigenetic modifiers, are more potent for silencing. The most commonly used silencing domain is the Krüppel-associated box (KRAB), which is one of the most potent natural repressors in the genome and used by half of all mammalian zinc finger transcription factors. Localizing KRAB to DNA can initiate heterochromatin formation by recruitment of complexes that may include the histone methyltransferase SETDB1 and the histone deacetylase NuRD complex [58–60]. In addition to silencing of promoters, KRAB has been shown to repress gene expression when targeted to distal and proximal gene regulatory elements like enhancers [30, 61–63].

**Fig. 1:**
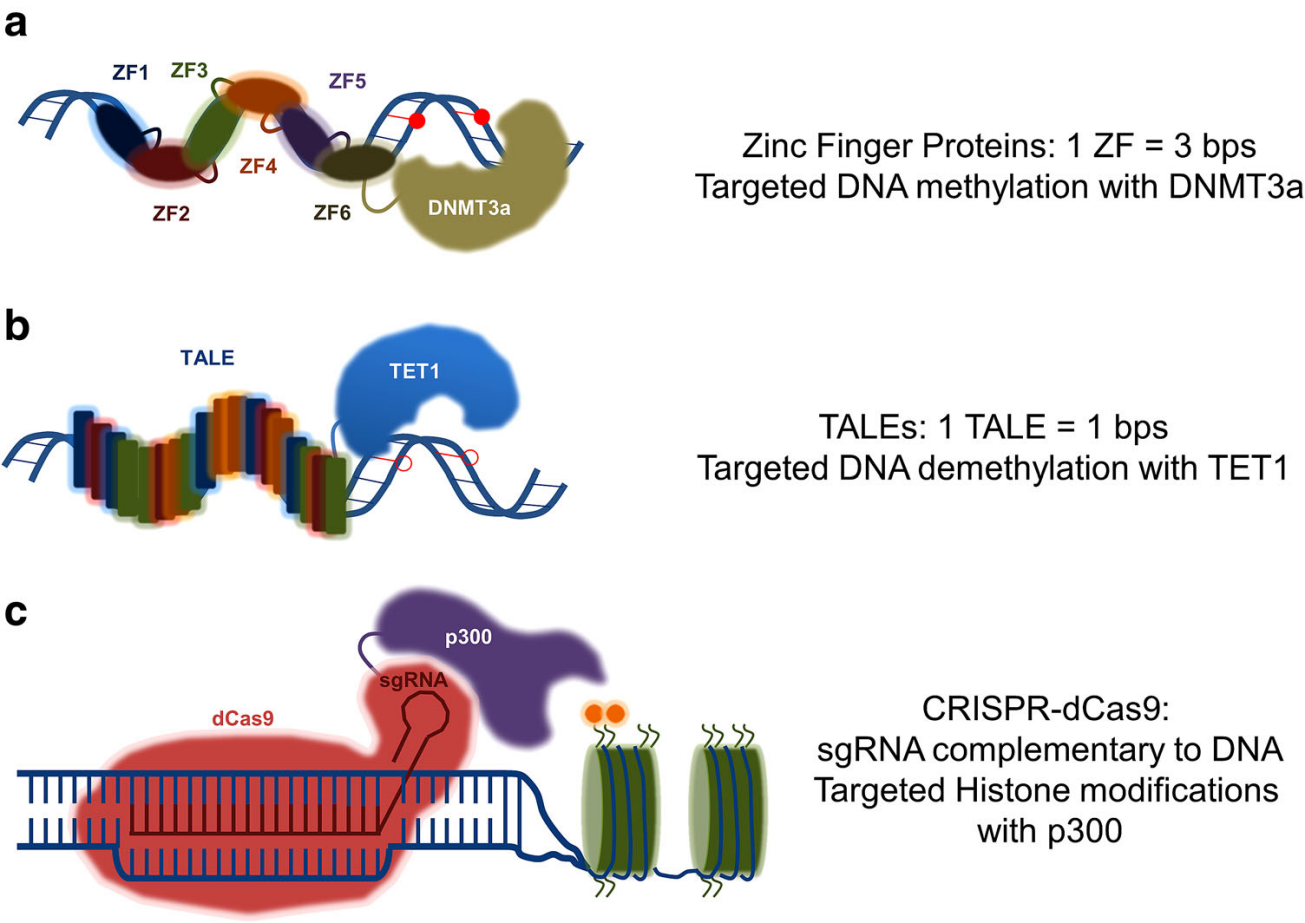
Epigenetic editing tools available. **a** Zinc finger proteins can recognize double-stranded DNA, fusion of 6 ZFPs can recognize an 18 bps sequence, and fused to a DNA methyltransferase like DNMT3a can add methylation to cytosine’s. **b** TALEs can recognize each module a single-base pair, fusion of several can recognize a locus, and fused to an oxidizing enzyme like TET1 can promote DNA demethylation. **c** CRISPR-dCas9 can bind to a sequence complementary to the sgRNA that is loaded with, and fused to a histone acetyltransferase like p300 can activate gene expression

Given the success of gene expression modulation by the use of artificial transcription factors, the possibility of using epigenetic modifications to manipulate the cellular machinery in a more sustained manner and to recruit writers or erasers to study the role of specific marks in different chromatin contexts was raised [18, 64]. Since epigenetic marks are inherited by daughter cells, the reprogramming might even be stable and maintained through cell divisions [6, 65]. The possibility to easily reverse epigenetic modifications in a targeted manner has opened new and exciting avenues for fundamental biological research. Indeed, the dynamic and reversible nature of the epigenetic modifications offers the possibility to reprogram any gene at will (Fig. 1). And that was how the epigenome editing field was born. Below we discuss the most used epigenetic effector domains in epigenetic editing (Table 1).

**Table 1:** Epigenetic effector domains used for targeted epigenetic editing

Gene regulation	Epigenetic effector	Enzymatic activity	Chromatin modification	Genes targeted
Repression	G9a	Methyltransferase	H3K9me2	*VEGF*-*A, Her2INeu, Fosb, E*-*Cadherin, Neruog, Grm2*
	Suv39h1	Methyltransferase	H3K9me3	*VEGF*-*A, Her2INeu, Neruog, Grm2*
	DNMT3 (A, A/L)	Methyltransferase	DNA methylation	*VEGF*-*A,* SOX2, *Maspin, EpCAM, CDKN2A, ARF, Cdkn1a,IL6ST, BACH2*
	LSD1	Demethylase	H3K4me2	*Gene enhancers*
	SIRT6, SIRT3	Deacetylase	H3K9ac	*Neruog, Grm2*
	KYP	Methylase	H3K9me1	*Neruog, Grm2*
	TgSET8	Methylase	H3K20me	*Neruog, Grm2*
	NUE	Methylase	H3K27me3	*Neruog, Grm2*
	HDAC8	Deacetylase	H4K8ac	*Neruog, Grm2*
	RPD3	Deacetylase	H4K8ac	*Neruoq, Grm2*
	Sir2a	Deacetylase	H4Kac	*Neruoq, Grm2*
	Sin3a	Deacetylase	H3K9ac	Neruog, Grm2
Activation	TET1	Deoxygenase	DNA demethylation	*ICAM*-*1, RHOXF2, BRCA1, RANKL, MAGEB2, MMP2*
	TET2	Deoxygenase	DNA demethylation	*ICAM*-*1, EpCAM*
	TET3	Deoxygenase	DNA demethylation	*ICAM*-*1*
	TDG	Glycosylase	DNA demethylation	*Nos2*
	p300	Acetylase	H3K27ac	*IL1RN, MYOD1, OCT4, HBE, HBG,ICAM*-*1*
	PRDM9	Methyltransferase	H3K4me3	*EpCAM,ICAM*-*1, RASSF1a, PLOD2*
	Dot1L	Methyltransferase	H3K79me	*EpCAM, PLOD2*

## Epigenetic Repression

The very first epigenetic modifier linked to a DNA-binding domain to establish epigenome editing was published in 2002 when an engineered ZF, designed to target the *VEGF*-*A* gene, fused to the histone methyltransferases G9a or SUV39H1 was able to show that H3K9 methylation is causative in *VEGF*-*A* gene repression [64]. It took a while before this study was followed by ZF-targeting the *HER2/neu* gene in cancer [66] and even in vivo by targeting the murine *Fosb* gene [67•]. Similarly, authors have fused a TALE, targeting the *E*-*Cadherin* gene, and dCas9, in combination with sgRNAs to target *VEGF*-*A*, to the SET domain of the histone methyltransferase G9a and demonstrated that this approach is effective in repressing genes, as seen with ZFPs [68, 69]. In the meantime, Zinc Fingers were also exploited in the first DNA methylation targeting studies by fusion to the catalytic domains of DNA methyltransferases Dnmt3a or including a fusion between Dnmt3a and Dnmt3L, which catalyze the *de novo* methylation of DNA. In these studies, the authors showed that targeted DNA methylation at gene promoters, of genes such as *VEGF*-*A* [70], *SOX2* and *Maspin* [71, 72•], and *EpCAM* [73], gene repression was achieved effectively. Similar results have been obtained by targeting the *CDKN2A* gene using a TALE fused to DNMT3A [74] as well as dCas9 using sgRNAs to target the *CDKN2A*, *ARF*, *Cdkn1a, IL6ST, and BACH2* genes, demonstrating the potency of epigenome editing [75, 76].

Currently, several engineered TALE domains as well as dCas9 proteins have also been fused to various histone modifiers. For example, for the catalytic domain of the LSD1 histone demethylase, authors were able to efficiently remove enhancer-associated chromatin modifications from targeted regions, without affecting control regions [61, 77]. Additionally, they found that removal of enhancer chromatin marks by these fusion proteins causes downregulation of proximal genes. Furthermore, using a set of 32 and 24 histone modifiers fused to TALEs targeting the *Neurog2* and *Grm2* genes, respectively, in combination with optogenetics for light induction, it was possible to assess the role of histone marks on the regulation of gene expression [78].

## Epigenetic Activation

In contrast to epigenetic repression, activation of epigenetically silenced genes has been more challenging. So far, only few active epigenetic marks have been addressed. The most common way to achieve gene re-expression has been done by using active DNA demethylation. ZFPs fused to the catalytic domain of TET1, TET2, and TET3 have been used to activate *ICAM1* gene expression, in a hypermethylated heterochromatic context, being TET2 the most efficient [79]. Alternatively, ZFPs have been used to enhance gene expression by fusion with the DNA demethylase thymidine DNA glycosylase (TDG) [80]. In other studies, researchers have fused the DNA demethylase TET1 to engineered TALEs targeting the *RHOXF2* gene, which led to the identification of the specific CpGs playing a role in gene expression [81]. Also, the CRISPR-dCas9 has also been fused to TET1 catalytic domain and was used to target the BRCA1 promoter, showing active DNA demethylation and gene upregulation [82]. Recently, a dCas9 system was further modified, by inserting two copies of bacteriophage MS2 RNA elements into the conventional sgRNAs, facilitating the tethering of the TET1 catalytic domain, in fusion with dCas9 or MS2 coat proteins, to target the *RANKL, MAGEB2,* or *MMP2* genes, and significantly upregulate gene expression, which was in close correlation to DNA demethylation of CpGs in their promoters [83]. Additionally, dCas9, TALEs, and ZFPs have been fused to the catalytic core of the p300 histone acetyltransferase to deposit H3K27ac and activate gene expression from promoters and distal enhancers [84•]. Recently, we have shown that induction of H3K4me3 as well as H3K79me, both marks are specific for active promoters, on silenced genes is enough to drive gene re-expression [42•].

## Next Stage of Epigenetic Editing: Sustained Epigenetic Reprogramming

Now that causality of epigenetic marks with respect to gene expression has been firmly proven, the next most fundamental question in epigenetic editing research is whether the newly introduced chromatin marks are stable and whether they are maintained over cell divisions. Indeed, the success of future clinical applications relies on longlasting epigenetic reprogramming. Only a few of the epigenetic editing studies have showed the mechanism of inheritance and stability of the epigenetic marks. The first studies concerned DNA methylation and H3K9 methylation for gene repression. On one hand, successful deposition of DNA methylation at the promoter of the *VEGF*-*A* gene caused effective silencing but, interestingly, the methylation and gene silencing were lost upon cessation of expression of the ZFP-fusion [85•]. On the other hand, another study showed that the induction of DNA methylation on the *MASPIN* tumor suppressor and *SOX2* oncogene resulted in stable silencing and was maintained through cell divisions [72•]. The differences in the results of these studies might be related to the different technical approaches (transient adenovirus infection vs. lentiviral insertion of inducible systems) and/or by the duration of the expression of the fusion proteins. Alternatively, these differential effects could be explained by the different chromatin contexts.

In an elegant paper, Bintu and colleagues used an artificial system to compare four repressive chromatin regulators that result in distinct chromatin modifications [86]: The EED protein of Polycomb repressive complex 2, which catalyzes H3K27 methylation; the KRAB domain that indirectly promotes H3K9 methylation; the DNMT3B that catalyzes DNA methylation; and the histone deacetylase 4 (HDAC4) enzyme. By transiently recruiting each protein for different periods of time they demonstrate that different types of repressed chromatins are generally associated with distinct time scales of repression. While DNA methylation shows a clear longstanding repression, histone deacetylation is less stable and has a fast recovery. Epigenetic editing studies are now required to confirm the general application of these findings for the various endogenous chromatin contexts.

While sustained gene repression by epigenetic enzymes seems conceptually more feasible, sustained gene activation is indeed poorly understood. In this sense, we have recently shown the different requirements to achieve longstanding gene re-expression that is maintained over time, depending on the chromatin microenvironment [42•]. While reactivation is achieved on hypomethylated promoters, hypermethylated promoters are less prone to sustained re-expression. Additionally, the requirement of histone post-translational modification crosstalk is an important event during reprogramming. H3K4me3 requires the presence of H3K79me in order to be stabilized and successfully maintained. Based on these, and other findings [87], it might turn out that the chromatin microenvironment greatly affects the outcome of epigenetic reprogramming.

## Clinical Applications and Future Perspectives

Aberrant gene expression due to epigenetic misregulation has been associated with several diseases, either as a symptom or even as a cause. The potency of epigenetic editing as a therapy is based on the reversible nature of epigenetic (mis)regulation [88]. In contrast to genetic mutations, epigenetic mutations thus allow for the possibility of reverting the abnormal patterns at a molecular level. Furthermore, site-specific epigenetic editing provides the opportunity to study the contributions of gene regulation to disease. The possible applications of epigenome editing can go as broad as from targeted reprogramming of cells via induced pluripotent stem cells to specialized cell types for clinical applications, to induction of genes involved in diseases with allelic imbalanced expression [89], and anticancer therapy.

Most of the focus so far has been placed on developing inhibitors of epigenetic enzymes, which act genome-wide and thus might suffer from side effects. The technology to activate endogenous genes by epigenetic rewriting of their own promoters allows physiological levels of expression, which likely resembles the natural conditions in normal cells better and is more specific than the small molecules inhibitors. The in vivo effectivity of the epigenetic editing approach has, for instance, been shown by the activation of glial cell line-derived neurotrophic factor (*GDNF*) using ZFPs in rat models, which resulted in protection against neural damage associated with Parkinson’s disease [90]. In this respect, activation of genes which compensate existence of mutated genes will allow the actual cure or at least the mitigation of the symptoms of diseases such as sickle cell anemia and β-thalassemia. For example, targeted activation of the developmentally silenced fetal γ-globin using ZFPs was achieved in mammalian cells, and could be used to counteract the loss of β-globin [91, 92]. In a pioneering study, researchers were able to activate multiple isoforms of *VEGF*-*A* with engineered ZFPs resulting in stimulation of functional angiogenesis in vivo, which was not achieved by exogenous overexpression of just one isoform [93]. Gene re-expression can also be used as a targeted therapy in cancer, as upregulation of silenced tumor suppressor genes is enough to induce cell death and inhibit cell migration, as proven by endogenous activation of several genes in cancer using ZFPs [33, 94, 95]. Additionally, engineered ZFP repressors have been designed to silence oncogenes and have been effective at slowing the growth of cancer cells not only in in vitro, but also in mouse models [30, 63, 72].

Although most of the mentioned studies have been done using transient transcriptional activators or repressors as effector domains, eventually, some of the findings are expected to be further optimized into therapeutic use by adopting epigenetic editing for such in vivo situations. There is already evidence that epigenetic editing therapy is feasible based on in vivo studies where targeting of the murine *Fosb* gene in the brain of living mice successfully controlled the drug response in regions of the brain harboring the reward system. In another study, targeting of *SOX2* promoter with ZFPs fused to DNA methyltransferases significantly delayed the tumorigenic phenotype of cancer cells in vivo and, importantly, the repression was stably maintained. Additional attention is currently given to aspects that require research in depth such as immunogenicity, cytotoxicity, off-target effects, and mode of delivery, in order to take these tools further into the clinic.

## Conclusions

Gene expression reprogramming can be achieved by targeted epigenetic editing of regulatory regions, and several DNA-binding platforms have been investigated for targeting various catalytically active epigenetic enzyme domains to multiple genes. The development of engineered DNA-binding domains has opened the possibility to address questions that were impossible to answer few years ago. Nevertheless, several aspects have to be addressed to fully exploit the approach for clinical applications, as delivery and sustainability are still an issue. Unraveling mechanisms for sustained gene re-expression necessitates the ongoing research into reinforcing epigenetic mechanisms depending on the chromatin microenvironment. Epigenetic editing can be used as a powerful research tool to study epigenetic molecular mechanisms as well as a biomedical tool toward a cure for what currently is incurable.
